# Advancing the Sustainable Development Goals through improving eye health: a scoping review

**DOI:** 10.1016/S2542-5196(21)00351-X

**Published:** 2022-02-25

**Authors:** Justine H Zhang, Jacqueline Ramke, Catherine Jan, Covadonga Bascaran, Nyawira Mwangi, João M Furtado, Sumrana Yasmin, Cynthia Ogundo, Miho Yoshizaki, Ana Patricia Marques, John Buchan, Peter Holland, Brandon A M Ah Tong, Jennifer R Evans, Nathan Congdon, Aubrey Webson, Matthew J Burton

**Affiliations:** International Centre for Eye Health, London School of Hygiene and Tropical Medicine, London, UK; Manchester Royal Eye Hospital, Manchester, UK; International Centre for Eye Health, London School of Hygiene and Tropical Medicine, London, UK; School of Optometry and Vision Science, University of Auckland, Auckland, New Zealand; Lost Child’s Vision Project, Taree, NSW, Australia; International Centre for Eye Health, London School of Hygiene and Tropical Medicine, London, UK; International Centre for Eye Health, London School of Hygiene and Tropical Medicine, London, UK; Department of Clinical Medicine, Kenya Medical Training College, Nairobi, Kenya; Division of Ophthalmology, Ribeirão Preto Medical School, University of São Paulo, Ribeirão Preto, São Paulo, Brazil; Sightsavers, Islamabad, Pakistan; International Centre for Eye Health, London School of Hygiene and Tropical Medicine, London, UK; Department of Ophthalmology, Mbagathi Hospital, Nairobi, Kenya; International Centre for Eye Health, London School of Hygiene and Tropical Medicine, London, UK; International Centre for Eye Health, London School of Hygiene and Tropical Medicine, London, UK; International Centre for Eye Health, London School of Hygiene and Tropical Medicine, London, UK; International Agency for the Prevention of Blindness, London, UK; The Fred Hollows Foundation, Melbourne, VIC, Australia; International Centre for Eye Health, London School of Hygiene and Tropical Medicine, London, UK; Centre for Public Health, Queen’s University, Belfast, UK; Centre for Public Health, Queen’s University, Belfast, UK; Zhongshan Ophthalmic Center, Sun Yat-sen University, Guangzhou, China; Permanent Mission of Antigua and Barbuda to the United Nations, New York, NY, USA; International Centre for Eye Health, London School of Hygiene and Tropical Medicine, London, UK; National Institute for Health Research Biomedical Research Centre; Moorfields Eye Hospital NHS Foundation Trust and UCLInstitute of Ophthalmology, London, UK

## Abstract

UN member states have committed to achieving the Sustainable Development Goals (SDGs) by 2030. This Review examines the published evidence on how improving eye health can contribute to advancing the SDGs (beyond SDG 3). We identified 29 studies that showed direct benefits from providing eye health services on SDGs related to one or more of poverty (SDGs 1, 2, and 8), education (SDG 4), equality (SDGs 5 and 10), and sustainable cities (SDG 11). The eye health services included cataract surgery, free cataract screening, provision of spectacles, trichiasis surgery, rehabilitation services, and rural community eye health volunteers. These findings provide a comprehensive perspective on the direct links between eye health services and advancing the SDGs. In addition, eye health services likely have indirect effects on multiple SDGs, mediated through one of the direct effects. Finally, there are additional plausible links to other SDGs, for which evidence has not yet been established.

## Introduction

In 2015, all UN member states committed to work towards achieving the Sustainable Development Goals (SDGs) by 2030.^1^ The 17 SDGs have broad objectives (panel), that include 169 targets and 232 indicators. They address many aspects of development, including poverty, hunger, health, education, gender equality, economic development, and environmental issues.

The latest global estimates for 2020, show that about 596 million people have distance vision impairment, of whom 43 million are blind.^2^ Most of this vision impairment could have been prevented or can be treated. A further 510 million people have unaddressed near vision impairment.^2^ Around 83% of vision impairment is found in low-income and middle-income countries. It is often concentrated in under-served groups within countries.^3^ Impaired eye health affects people across the full life course, represents a major public health challenge, and is a substantial barrier to sustainable development.

This Review forms part of the *Lancet Global Health* Commission^4^ on Global Eye Health, which defined eye health as the state when vision, ocular health, and functional ability are maximised, thereby contributing to overall health and wellbeing, social inclusion, and quality of life.^5^

We hypothesised that eye health services that improve vision and functional ability can, in turn, lead to the advancement of multiple SDGs. We consider eye health services to include all types of interventions that improve eye health, encompassing the spectrum of promotion, prevention, treatment, and rehabilitation.^6^ Potentially, there is a two-way relationship between eye health and the SDGs; however, here we are primarily concerned with the impact that improved eye health services could have on the SDGs, rather than the impact that improvements in SDG-related areas can have on eye health. We summarise the nature and extent of published evidence that services improving eye health contribute to advancing specific SDGs and identify the main pathways by which such services lead to advancement of the SDGs.

## Methods

We anticipated the literature on the relationship between the SDGs and eye health to be broad, complex, and very heterogeneous in nature. Therefore, a scoping review method was selected as the most appropriate approach to identify and map the available evidence.^7^ We report the Review in accordance with the PRISMA Extension for Scoping Reviews (appendix, pp 2–3).^8^

To guide the review, we initially asked Commissioners of the *Lancet Global Health* Commission^4^ on Global Eye Health to review all 169 SDG targets and outline possible links between eye health services and specific SDG targets.^1^ After reviewing the suggested links, a logic model was developed and iteratively refined by the authorship group, and is published in the protocol.^9,10^ This model was used to inform our search strategy.

A protocol for this scoping review was registered prospectively with Open Science Framework (gu4z6) on Nov 15, 2019, and published.^10^ As this study only included published data, ethics approval was not sought.

## Search strategy and selection criteria

On Oct 31, 2019, we searched MEDLINE, Embase, and Global Health using a search strategy developed by an experienced information specialist from Cochrane Eyes and Vision (the MEDLINE search strategy is included in the appendix; pp 4–6). To identify further potentially relevant studies, we examined reference lists of all included articles. We also provided a list of the included studies to relevant Commissioners and requested they identify further potentially relevant studies for consideration in the review.

All primary research studies or meta-analyses were included if they reported the relationship between an eye health service and either an outcome related to one of the SDGs, or an element on a pathway between eye health and an SDG. A list of indicative pathway elements agreed on by the authorship group of this Review can be found in the appendix (p 7). Systematic reviews without meta-analyses were excluded.

We recognise that impaired eye health has many consequences for other health and wellbeing outcomes in SDG 3. Therefore, the *Lancet Global Health* Commission on Global Eye Health^4^ has also undertaken complementary reviews (published separately) investigating links between eye health and other health and wellbeing outcomes.^11–14^ Therefore, for the purposes of this Review, we excluded studies with SDG 3-related health and wellbeing outcomes.

Further criteria were established during the review process. Studies were excluded if there was no comparison group, or the study only compared different types of eye treatments against each other (eg, eye drop A *vs* eye drop B). We excluded these studies because without a comparison group, identifying whether the study findings were due to the effects of the eye health intervention or due to some other factor would not be possible, and studies comparing different treatments were unlikely to answer the question of whether any particular eye health intervention affected an SDG-related outcome. Studies were also excluded if simulation was used in the exposure group (eg, using goggles to simulate the effects of an eye condition) or the outcome (eg, virtual reality driving simulators), since this method was deemed to be insufficient for assessing the real-life effect of eye health services on the SDGs. Excluded studies also included those with a sample size of less than 100 participants. We excluded studies with small sample sizes post-hoc, as such studies would be unlikely to contribute to the aims of this scoping review. These studies are summarised in the appendix (pp 10–11).

Studies from all time periods were eligible for inclusion. We included studies from any world region (classified according to the seven Global Burden of Disease super-regions). No language restrictions were used. All potentially relevant publications in languages other than English were translated into English or screened and extracted by someone with at least professional working proficiency in that language. We included published peer-reviewed manuscripts only. As this scoping review was concerned with identifying the extent of evidence in published literature, grey literature was not searched.

## Selection of sources of evidence

Titles and abstracts were independently screened by two investigators with web-based review management software (Covidence). Full texts were then independently screened by two investigators to establish eligibility for inclusion. Any conflicts were resolved with a third reviewer.

## Data charting and data items

Data charting forms were developed with Google Forms and were pilot-tested by nine investigators (JHZ, JR, CJ, CB, NM, JMF, SY, CO, and MY) on two studies. A copy of the Google Form used for data extraction, which lists the data items that were collected, can be found in the appendix (pp 8–9). Two investigators charted the data of included studies, working independently, for all data items except for the type of study (eg, randomised controlled trial and prospective cohort study), which was charted by a single epidemiologically-trained investigator for consistency and verified by another investigator. Randomised controlled trials were explicitly indicated in the results tables. Countries of study were mapped to Global Burden of Disease super-regions (hereafter referred to as regions) by a single investigator. In the case of unclear information during data charting, we planned to contact authors directly, but this step was not required. We did not plan to formally appraise the quality of individual sources of evidence.

## Synthesis of results

Following data charting, results were synthesised by mapping the retrieved evidence to our eye health–SDG logic model (appendix p 9).^10^ Closely linked SDGs (eg, SDG 1 no poverty, SDG 2 zero hunger, and SDG 8 decent work and economic growth) were grouped together, and evidence for SDG-related outcomes (eg, household income) were synthesised under these umbrella SDG groups.

Each pathway from exposure to outcome and the effect of the eye care service (which resulted in a change in eye health) on the SDG-related outcome was examined separately. Relevant evidence for each pathway was collated and summarised, including effect estimates when available. The directionality and extent of evidence supporting each pathway in the summary figure was indicated by differing arrow widths and colours. We planned to develop separate protocols for meta-analysis if sufficient homogenous studies were found for individual exposure–outcome pathways; however, this synthesis was not possible for any pathway.

## Results

**Figure 1:**
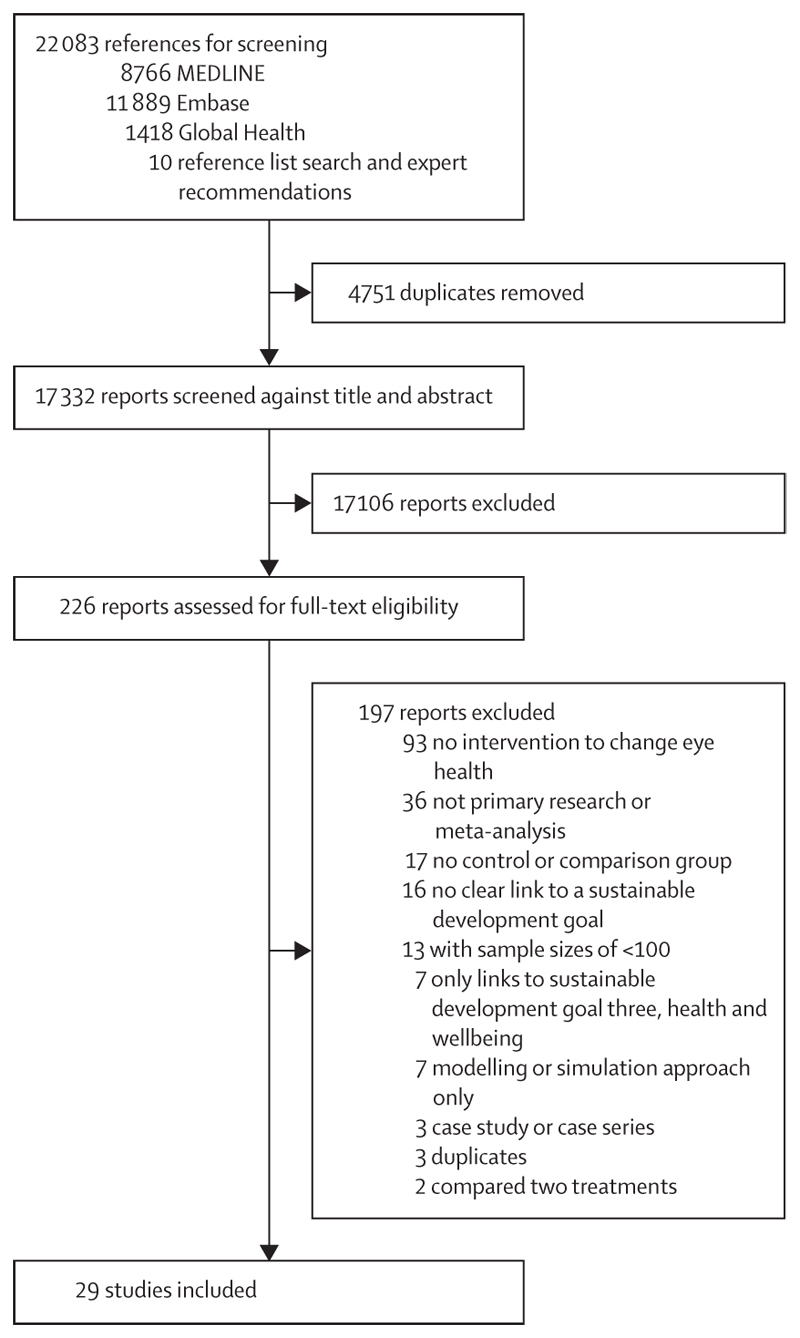
Study selection

The search returned 17 332 unique publications. Titles and abstracts were assessed for eligibility, and 226 were selected for full-text assessment (including ten studies identified through reference list searches and expert recommendations); four full texts were not in English and were assessed by someone with at least a professional working proficiency in the language. 29 studies met the inclusion criteria and are considered in this Review in detail (figure 1). There were a further 13 studies that met all criteria except having a sample size of at least 100 participants. We provide summaries of these 13 studies in the appendix (pp 10–11).

All included studies were in English. Study size ranged from 185 participants to 559 546 participants (median 1200, IQR 440–4067). The majority of studies were observational in design, and only six studies (21%) were randomised controlled trials (table 1).

Several studies were conducted in more than one region (table 1). Most studies were done in the high-income region (19 studies, 66%). The regions of (1) sub-Saharan Africa, (2) south Asia, and (3) southeast Asia, east Asia, and Oceania each had nine included studies (31%). Only one study was done in the north Africa and Middle East region. Two regions had no included studies (one region being Latin America and the Caribbean and the second being central Europe, eastern Europe, and central Asia).

We mapped the studies to individual SDGs or umbrella SDG groups. The largest proportion of included studies (12 studies, 41%) were mapped to poverty-related SDGs (1, 2, and 8), followed by education (SDG 4; nine studies, 31%), sustainable cities (SDG 11; six studies, 21%), and equality (SDGs 5 and 10; four studies, 14%).

For the poverty-related SDGs (1, 2, and 8), studies show that the main pathways by which eye health services contribute to the advancement of these SDGs are through improvement in one or more of: workplace productivity, household per capita expenditure, household income, employment rates, and economic productivity. For education (SDG 4), eye health services were found to contribute to improved academic test scores. For equality (SDGs 5 and 10), eye health services eliminated gaps in per capita expenditure. For sustainable cities (SDG 11), eye health services were found to reduce driving-related difficulties and motor vehicle crashes.

We did not identify any eligible studies that mapped directly to outcomes related to the environment and energy (SDGs 7 and 12–15), peace and partnership (SDGs 16 and 17), water and sanitation (SDG 6), or innovation (SDG 9).

The key findings of the 29 included studies are briefly summarised in table 2. A more detailed synopsis of methods and findings for each study is provided in the appendix (pp 12–16). 27 studies reported that eye health services had a positive effect on advancing one or more SDG targets. Two studies reported a negative effect on SDG-related outcomes, though their findings were mixed or inconclusive. One of these studies showed that eye health improved at the aggregate level but inequality increased,^37^ whereas the other study showed that legally blind adults who attended specialised schools, for people with vision impairment, had a lower salary compared to legally blind adults who attended public schools.^21^

However, the authors of this study acknowledged that this association could have been confounded by other determinants (eg, the public school group had more usable residual vision than the specialised school group); further, braille literacy was better in the specialised school group than the public school group.

**Figure 2:**
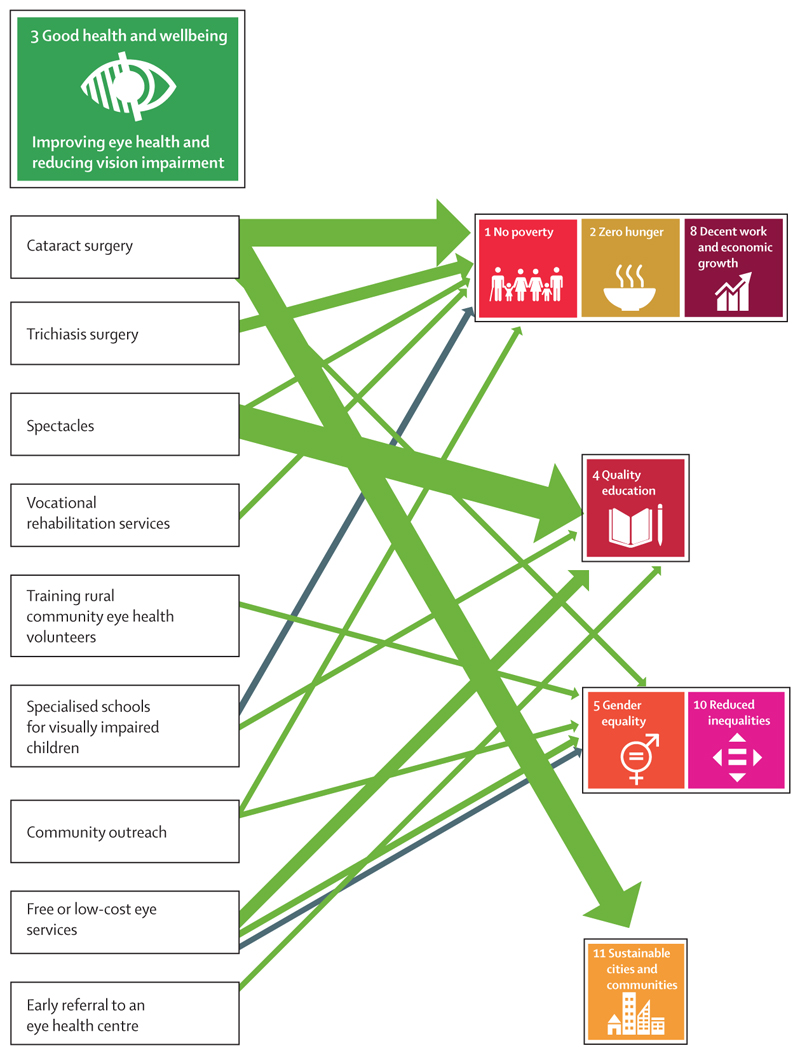
Summary of evidence linking specific services to improve eye health and specific Sustainable Development Goals Green arrows indicate a direct positive benefit (n=27); grey arrows indicate a negative relationship (n=2). The width of the arrows represents the number of studies.

We mapped out the reported direct connections or pathways between specific eye health services and the relevant SDG for the 29 included studies (figure 2; appendix p 17). The range of eye health services considered was broad, and included cataract surgery, free cataract screening, provision of spectacles, trichiasis surgery, rehabilitation services, and rural community eye health volunteers. Cataract surgery and spectacles were the interventions with the largest number of reported beneficial effects on an SDG.

## Discussion

**Figure 3:**
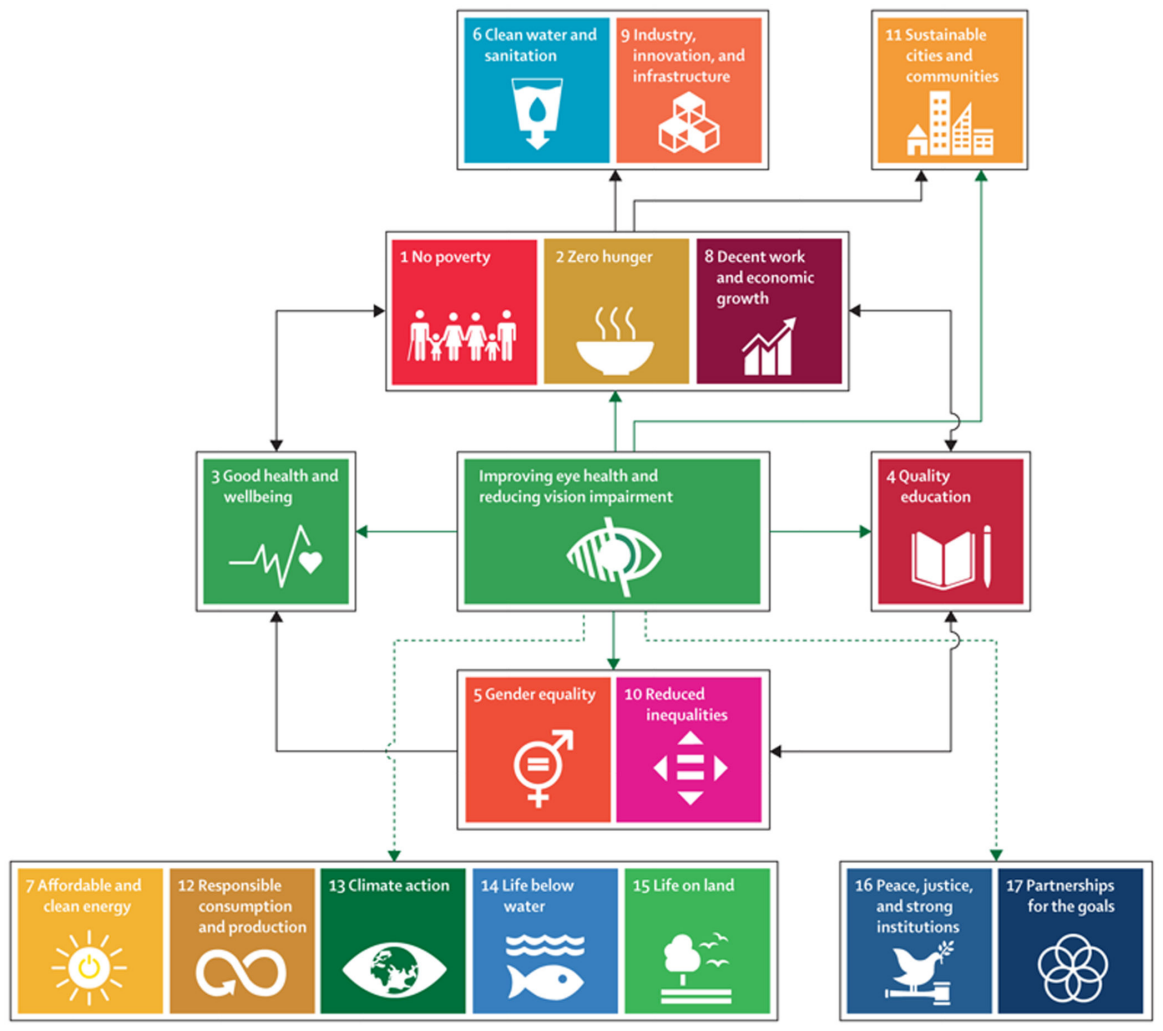
Improving eye health Solid green arrows indicate relationships with direct evidence of a beneficial effect, black arrows represent likely indirect effects, and dashed green arrows represent hypothesised beneficial effects.

We identified 29 studies that reported direct links between eye health services or interventions and their largely beneficial effects on SDGs related to poverty (SDGs 1, 2, and 8), education (SDG 4), equality (SDGs 5 and 10), and sustainable cities (SDG 11). Our findings expand on the known associations between vision impairment and SDG-related outcomes, through providing a comprehensive perspective on the links between eye health services and advancing several of the SDGs. In addition to the direct links we identified, eye health plausibly has several indirect effects on the same and additional SDGs; for example, improved eye health promotes educational outcomes in girls and boys alike, thus improving gender equality (an indirect effect on SDG 5). Finally, there are additional hypothetical links to other SDGs, for which evidence is currently absent. We have represented all three types of relationship in figure 3 and go on to discuss each in turn.

### Zero poverty and hunger; decent work and economic growth (SDGs 1, 2, and 8)

A strong association between poverty and vision impairment has been reported in many settings.^44–46^ This relationship is likely to be a bidirectional relationship, with poverty both a cause and a consequence of poor eye health. Poverty and low socioeconomic status can result in reduced access to quality eye health services, delayed diagnosis, and limited access to treatment.^47^ Poverty is also strongly associated with worse general health, which might adversely affect eye health. For example, poor nutrition leading to vitamin A deficiency results in a progressive eye disease called xerophthalmia.^48^

Conversely, poor eye health and social exclusion of people living with vision impairment might lead to poverty. Several studies, including the World Health Survey, have found that people with vision impairment were less likely to be employed^49^ and have lower salaries if employed than those without vision impairment,^50^ reducing household income. Moreover, there can be additional lost income for household members who need to stay home to care for someone with vision impairment.^51^ Other highly symptomatic eye conditions, which might not reduce visual acuity (eg, some cases of dry eyes), could also affect employment.^52,53^ The Commission^4^ estimated annual global economic productivity losses were equal to US$411 billion in purchasing power parity in 2018. Finally, some eye problems can lead to stigma. For example, a study in the USA found that digitally altered photographs of the same person from typical eye alignment to misalignment (squint or strabismus) reduced job hiring scores in women.^54^

We found evidence that interventions to improve vision and functional ability reduce poverty and improve economic prospects (table 2).^15–26^ For example, provision of free spectacles to tea workers with presbyopia in India improved workplace relative productivity.^15^ Prospective cohort studies showed increases in household per capita expenditure in people with vision impairment who underwent cataract surgery.^16,17^ Implementing effective eye health services would be a way to break the negative cycle of poor eye health, which leads to poverty, leading to worse eye health, and thus more poverty.^55^ The effects of improved eye health cascades beyond poverty reduction, to achieve wider benefits, such as improved health and wellbeing (SDG 3),^56,57^ and education (SDG 4).^58^

### Improving health and wellbeing (SDG 3)

In this Review we specifically excluded links between eye health and health and wellbeing (SDG 3). Complementary reviews undertaken by the *Lancet Global Health* Commission on Global Eye Health^4^ explore the inter-section between eye health and other health and wellbeing outcomes, including the associations between vision impairment and mortality,^11^ vision impairment and falls,^12^ vision impairment and quality of life,^13^ and vision impairment and dementia, mental health, cardiovascular disease, respiratory disease, and cancer. A complementary scoping review investigating the prevalence and impact of dual sensory impairment has also been done.^14^

### Improving education outcomes (SDG 4)

Good vision is associated with improved educational outcomes.^59,60^ A study of almost 1 million children across 30 low-income and middle-income countries found that children with vision impairment were two to five times less likely to be included in formal education compared with children without a disability.^61^ In high-income countries, although school attendance is generally mandatory, educational scores tend to be poorer in children with vision impairment than children without.^62^

We examined this relationship by investigating which eye health services are important for improving educational outcomes, and identified five randomised controlled trials, which were all related to the provision of spectacles to children.^21,27–34^ This inexpensive, simple intervention has been shown to improve academic test scores and literacy skills, which in turn can improve future opportunities for decent work and paid employment.

### Reducing inequality (SDGs 5 and 10)

Poor eye health disproportionately affects low resourced countries and disadvantaged groups within countries. We found a few studies showing that eye health services reduce inequity by gender,^35,36^ and improve socioeconomic status (measured as increased household per capita expenditure).^17^ These findings are a promising start and there is scope for further research in this area.

One study in Scotland showed that offering free eye examinations actually widened inequalities across income and education groups.^37^ This example of an intervention-generated inequality is not uncommon with universal interventions designed to be accessed by everyone in the same way, as more advantaged (low-risk) groups are more able to access and benefit from the intervention.^63^ The Scottish study highlights the need to avoid intervention-generated inequalities, so that no one is left behind in the pursuit of the SDGs. Targeting services to those with the most to gain is one way to reduce inequalities. An example would be increasing the cataract surgical rate in rural areas, where a higher proportion of people tend to have vision impairment from cataract compared with people in urban areas.^64^ Another promising strategy to promote equity in the pursuit of the SDGs is proportionate universalism, which combines universal and targeted approaches, and aims to improve health for everyone while targeting underserved groups so that the degree of health improvement is proportionate to the level of disadvantage.^65^ We are unaware of any documented examples of proportionate universalism in eye health, although we anticipate this lack of evidence will change in the universal health coverage and SDG era.

### Sustainable cities and communities (SDG 11)

Vision impairment can reduce driving safety and increase motor vehicle collisions,^66^ thereby affecting SDG target 11.2, which aims to provide access to safe, affordable, accessible, and sustainable transport systems for all. Vision impairment is not simply limited to visual acuity, but also includes visual field and colour vision problems. A complementary review undertaken by the *Lancet Global Health* Commission on Global Eye Health^4^ further explores the intersection between eye health and driving safety.^67^ The review determined that some causes of vision impairment, such as glaucoma and cataract, are associated with motor vehicle collisions and unsafe driving practices. Cataract has been found to be associated with approximately 2·5-fold increased odds of motor vehicle collisions,^68^ and glaucoma, which causes visual field loss, has been found to be associated with 1·65 times greater rates of motor vehicle collisions than in people without glaucoma.^69^

Several studies included in this Review show cataract surgery reduced driving-related difficulties and motor vehicle collisions.^38–43^ Most countries have a legal threshold of visual acuity that must be achieved in order to drive;^70^ eye health services helping to achieve this visual acuity threshold would be anticipated to contribute to improving road safety.

## The remaining SDGs

Although the evidence identified for direct links between eye health services and the SDGs was limited to SDGs 1–5, 10, and 11, there are plausible indirect links between providing eye health services and the advancing of other SDGs. These indirect links, mediated through other SDGs, are harder to assess and attribute to improvements in eye health compared to direct links (figure 3).

### Clean water and sanitation (SDG 6)

Eye health services could have an indirect effect on clean water and sanitation, through reducing poverty, which in turn is linked to improved infrastructure for water, sanitation, and hygiene.^71^ Conversely, improvements in the provision of clean water and sanitation are important in trachoma control, the most common infectious cause of blindness.^72^

### Planetary health (SDGs 7 and 12–15)

Globally, health care is estimated to contribute about 5% of the world’s total greenhouse gas emissions.^73,74^ Ophthalmology is a major component of these emissions. For example, cataract surgery is one of the most common surgical procedures globally, and in the UK, ophthalmology has more outpatient attendances than any other hospital speciality.^75^ Clinical activity is forecast to rise by 50% over the next 20 years due to growing and ageing populations.^76^ SDG 13 requires urgent action to combat climate change and its impacts. The evidence on the effects of eye care on this SDG is notable by its absence.

The eye health sector has a responsibility towards environmental sustainability, as does any other sector in society. There are environmental impacts from the manufacturing, running, and disposal of eye health equipment, disposables, and drug treatments. Eye care services in high-income countries generate large amounts of waste products from clinics and surgeries, such as plastic single use containers for eye drops, or cataract surgical packs containing equipment and pharmaceuticals that are partly or totally unused and subsequently discarded; once sterile products are opened, local protocols might prevent the contents being resterilised and reused for other patients.^77^ Moreover, the comparison with services in lower-income and middle-income country settings has shown that the carbon footprint of one phacoemulsification cataract operation in an Indian institution is just 5% of that of the same procedure in the UK.^78^

There is minimal published research regarding how eye health affects planetary health. During abstract screening for this scoping review, we found three studies (not meeting our eligibility criteria) that discussed the carbon footprint of cataract surgery; these studies concluded that there is a need for further study in this area,^79,80^ and that phacoemulsification cataract surgery has a larger carbon footprint than modified small incision cataract surgery at two Scottish centres.^81^ Eye health services inevitably have effects on the environment, but how and what we can do to minimise the negative impact on the environment is an area that urgently needs to be addressed. The *Lancet Global Health* Commission on Global Eye Health^4^ has also conducted a scoping review^82^ on the environmental sustainability of eye health-care delivery.

The association between eye health and planetary health is probably bidirectional. As the focus of this Review is on how eye health services affect the SDGs, we did not search for evidence in the opposite direction regarding how planetary health interventions could improve eye health (eg, housing improvements might reduce risk of infectious eye conditions). However, these effects are important areas to consider for future work.

### Industry, innovation, and infrastructure (SDG 9)

Our Review did not identify any studies with a direct link between eye health and SDG 9. However, given that improved eye health promotes educational outcomes (SDG 4), and a highly skilled workforce contributes to industry and innovation, indirect links between eye health and SDG 9 are conceivable.

### Peace and partnership (SDGs 16 and 17)

This Review did not identify any studies with a direct link between eye health and SDGs 16 or 17. The global eye health community has developed some excellent examples of international partnership, including the International Trachoma Initiative,^83^ and the African Programme for Onchocerciasis Control.^84^ These partnerships both led to substantial reductions in the global burden of major eye diseases whose spread across borders could not have been addressed in any other way. Partnerships between public and private organisations have also been valuable, for example, in Timor-Leste a public–private partnership was successful in setting up a national spectacle programme.^85^ In 1987, Merck & Co made a groundbreaking donation of ivermectin for the onchocerciasis control programme, which arguably stimulated other public–private partnerships in later years.^86^

## Strengths and limitations

To our knowledge, this paper is the first scoping review to explore how eye health services contribute to the advancement of the SDGs. Published peer-reviewed manuscripts were comprehensively assessed with no language, time period, or geographical restrictions. We included studies that described the links between eye health services and the SDGs, and not those that reported vision impairment without mention of eye health services. This pragmatic choice made abstract screening feasible given the broad search strategy we applied.

Our review method might have omitted some research relevant to eye health and the SDGs. Studies in which the SDG-related outcome of interest to this Review was not reported in the abstract were not included. We also omitted evidence from grey literature, in which indexing of primary studies is poor: this approach could have led to the exclusion of some reports from governmental and non-governmental organisations. Finally, as this Review was a scoping review, we did not perform a formal quality assessment of studies, nor did we do an overall assessment of the strength of the evidence for each observed association. We anticipated that the studies would be heterogenous and set out to identify and map the available evidence.

## Recommendations for future research

We believe that improving understanding among the global community of how and in what ways eye health services affect wide-ranging societal issues across all SDGs is important. We found only 29 studies meeting inclusion criteria, indicating that relatively little research has been done on exploring the effect of eye health services on broad societal concerns such as poverty. Although direct effects do not exist between eye health and all SDGs, there is a disconnect between what the global community should know, and what we do know, about the effect of different eye health services on relevant SDGs. This gap should be addressed in future eye health research, including high-quality observational studies, quasi-experiments, and randomised controlled trials as appropriate. In addition, quantifying and comparing the relative impact of different interventions would be useful.

We found only one other systematic or scoping review looking at connections between improvement in a specific area of health and the SDGs: physical activity promotion strategies and their effect on the SDGs.^87^ The authors of that scoping review found that physical activity promotion had wide-ranging effects on the SDGs beyond SDG 3, but also concluded that “not all plausible links… are currently supported by scientific evidence, highlighting important research gaps”.^87^

Promoting equity should be a central pillar to all interventions, and we recommend that all future studies investigating the effect of an intervention should inspect equality dimensions across gender, socioeconomic status, and place of residence as a minimum. Embedding equity in this way is even more pertinent to studies investigating preventive interventions, as discussed earlier. For the global community to risk introducing interventions that widen inequalities in society would be a step backwards.

## Recommendations for policy

Eye health does not currently feature within the 169 targets and 232 indicators of the SDG monitoring framework. This Review has highlighted direct and indirect effects of improving eye health on advancing multiple SDGs. As such we think that there is a strong case that improving eye health is a powerful enabling tool for sustainable development and should receive political prioritisation and financial support commensurate with its broad relevance. There is a need for embedded policies and interventions to improve eye health in multiple sectors including education, workplaces, and social services. Eye health needs to be included in the health mainstream as part of universal health coverage.

## Conclusions

We have reviewed the evidence showing that eye health services aimed at maximising vision, ocular health, and functional ability have broad benefits and potentially promote the advancement of multiple SDGs, in particular, reducing poverty, supporting economic opportunities, and improving educational outcomes. This evidence supports the case for investing in eye health services, leading to cascading beneficial effects to widespread societal needs embodied by the SDGs.

## Supplementary Material

Supplementary Material

## Figures and Tables

**Table 1:** Characteristics of the 29 included studies

	Studies, n (%)
**SDG** *	
Poverty-related (SDGs 1, 2, and 8)	12(41%)
Education (SDG 4)	9 (31%)
Equality (SDGs 5 and 10)	4 (14%)
Sustainable cities (SDG 11)	6 (21%)
Environment (SDGs 7 and 12-15)	0
Peace and partnership (SDGs 16 and 17)	0
Water and sanitation (SDG 6)	0
Innovation and industry (SDG 9)	0
**Year of publication**	
1998–2004	5 (17%)
2005–09	2 (7%)
2010–14	11 (38%)
2015–19	11 (38%)
**Study design**	
Meta-analysis	2 (7%)
Randomised controlled trial	6 (21%)
Prospective cohort study	9 (31%)
Retrospective cohort study	5 (17%)
Pair or series of cross-sectional studies	2 (7%)
Exposure cross-over study	1 (3%)
Economics study	4 (14%)
**Global Burden of Disease super-region** †	
High-income country	19 (66%)
Southeast Asia, east Asia, and Oceania	9 (31%)
South Asia	9 (31%)
Sub-Saharan Africa	9 (31%)
North Africa and the Middle East	1 (3%)
Latin America and the Caribbean	0
Central Europe, eastern Europe, and central Asia	0
**Funding**	
Public	7 (24%)
Private, charity, or industry-sponsored	10 (34%)
Mixed (public and private)	4 (14%)
Not reported	8 (28%)

SDG=Sustainable Development Goal.

* We excluded studies reporting outcomes related to SDG 3; includes two studies that are linked to two different SDG groups.

† Several studies are linked to >1 Global Burden of Disease super-region. The percentages are based on a denominator of 29.

**Table 2:** Summary of the influence that interventions to improve eye health have on the advancement of SDGs, by SDG groups of poverty, education, equality, and sustainable cities

Summary of study findings	
Poverty-related: SDG 1 no poverty; SDG 2 zero hunger; and SDG 8 decent work and economic growth	
Workplace relative productivity (one study)	A randomised controlled trial showed that provision of free spectacles to tea workers with presbyopia in India improved workplace relative productivity by 22% (p<0·0001)^15^
Household per capita expenditure (two studies)	Prospective cohort studies showed increases in household per capita expenditure in people with vision impairment who underwent cataract surgery;^16,17^ for example, in the Philippines, household per capita expenditure increased by 88% over 1 year in people who underwent cataract surgery (p<0·0001)
Household income (four studies)	Several prospective cohort studies showed that household income increased after cataract surgery;^18–20^ for example, 1 year after provision of cataract surgery in marginalised communities in rural India, the proportion of households with a monthly income <1000 rupees decreased from 51% to 21% (p=0·05)
••	One retrospective cohort study showed that, in people who became legally blind by the age of 6 years in the USA, those who attended specialised schools for people with vision impairment had a lower salary than those who attended public schools, although this difference could have been confounded by other determinants^21^
Employment rates (one study)	A retrospective cohort study showed that some vocational rehabilitation services for people with vision impairment in the USA were positively associated with paid employment; for example, training and support services were associated with increased odds of obtaining paid employment (odds ratio 1·10; p=0·001)^22^
Economic productivity (four studies)	Cost-effectiveness and cost-evaluation studies showed benefits to economic productivity from cataract surgery^23,24^ and trichiasis surgery;^25,26^ for example, one study showed that there was a net 13 year US$123·4 billion return on investment from a 1 year cohort of patients who had had cataract surgery, which included an increase in US national productivity of $25·4 billion^23^
**SDG 4 quality education**	
Academic test scores (seven studies)	Five randomised controlled trials showed that provision of spectacles to children improved academic test scores,^27–31^ and this finding was also seen in prospective cohort studies;^32,33^ for example, a study in China showed that vision correction with spectacles reduced the odds of failing a class by 44% (p<0·01)^28^
Reading ability (two studies)	Cohort studies found improved letter identification scores with spectacle wear^34^ and improved reading ability with attendance at specialised schools^21^
**Equality: SDG 5 gender equality and SDG 10 reduced inequalities**	
Gender inequality (two studies)	A systematic review and meta-analysis found reduced gender inequality in all-cause blindness, clinic attendance, cataract surgery coverage, and trachoma treatment coverage as a result of interventions to promote eye service use supported by trained rural community eye health volunteers in lower-income and middle-income countries^35^
••	A pair of cross-sectional surveys showed that free cataract screening and low-cost, high-quality cataract surgery in China resulted in a reduction in gender disparity in willingness to pay when comparing 5 year follow-up (88% willingness to pay in men and 91% in women) to baseline (67% in men and 50% in women)^36^
Equity, as measured by per capita expenditure (one study)	A cohort study showed that people who had cataract surgery in Kenya, the Philippines, and Bangladesh were poorer than non-visually impaired people before they had their surgery (p≤0·02) but after surgery, there was no longer a difference in per capita expenditure between the cataract group and the non-visually impaired group (p≥0·20), showing that equity as measured by per capita expenditure improved^17^
Inequalities in use of eye care services (one study)	A series of annual cross-sectional surveys showed that free eye examinations in Scotland increased use of eye care services at the aggregate level, but widened inequalities by income (p<0·001) and education (p<0·001)^37^
**SDG 11 sustainable cities and communities**	
Driving-related difficulties (one study)	A meta-analysis showed reduced driving-related difficulties after cataract surgery (pooled odds ratio 0·12; 95% CI 0·10–0·16)^38^
Motor vehicle crashes (five studies)	Several observational studies showed that cataract surgery reduced motor vehicle crashes (with all studies reaching significance)^39–43^

The relationship between eye health and SDG 3 is discussed elsewhere in complementary reviews.^11–14^ The table includes two studies that are linked to two different SDG groups. The full list summarising all included studies can be found in the appendix (pp 12–16). SDGs=Sustainable Development Goals.

## Data Availability

Data generated from this Review will be available upon reasonable request from Justine H Zhang (justine.zhang@lshtm.ac.uk).

## Panel Summary of the Sustainable Development Goals (SDG)

- SDG 1: end poverty in all its forms everywhere
- SDG 2: end hunger, achieve food security and improved nutrition, and promote sustainable agriculture
- SDG 3: ensure healthy lives and promote wellbeing for all at all ages
- SDG 4: ensure inclusive and equitable quality education and promote lifelong learning opportunities for all
- SDG 5: achieve gender equality and empower all women and girls
- SDG 6: ensure availability and sustainable management of water and sanitation for all
- SDG 7: ensure access to affordable, reliable, sustainable, and modern energy for all
- SDG 8: promote sustained, inclusive, and sustainable economic growth, full and productive employment, and decent work for all
- SDG 9: build resilient infrastructure, promote inclusive and sustainable industrialisation, and foster innovation
- SDG 10: reduce inequality within and among countries
- SDG 11: make cities and human settlements inclusive, safe, resilient, and sustainable
- SDG 12: ensure sustainable consumption and production patterns
- SDG 13: take urgent action to combat climate change and its impacts
- SDG 14: conserve and sustainably use the oceans, seas, and marine resources for sustainable development
- SDG 15: protect, restore, and promote sustainable use of terrestrial ecosystems, sustainably manage forests, combat desertification, halt and reverse land degradation, and halt biodiversity loss
- SDG 16: promote peaceful and inclusive societies for sustainable development, provide access to justice for all, and build effective, accountable, and inclusive institutions at all levels
- SDG 17: strengthen the means of implementation and revitalise the global partnership for sustainable development
