# Supplementary material for: Advancing the Sustainable Development Goals through improving eye health: a scoping review
Source: Lancet Planet Health. Author manuscript; Available in PMC 2025 Oct 23. (PMC7618288; doi:10.1016/S2542-5196(21)00351-X)
Supplement: Supplementary Material [file EMS209640-supplement-Supplementary_Material.pdf]

THE LANCET
Planetary Health

Supplementary appendix

This appendix formed part of the original submission and has been peer reviewed. We post it as supplied by the authors.

Supplement to: Zhang JH, Ramke J, Jan C, et al. Advancing the Sustainable Development Goals through improving eye health: a scoping review. *Lancet Planet Health* 2022; published online Feb 23. https://doi.org/10.1016/S2542-5196(21)00351-X.

Supplementary Appendices

Supplement to: Zhang JH, Ramke J, Jan C, et al. Advancing the Sustainable Development Goals through improving eye health: a scoping review.

Contents

Item	Page
Appendix 1: Preferred Reporting Items for Systematic reviews and Meta-Analyses extension for Scoping Reviews (PRISMA-ScR) Checklist	2
Appendix 2: MEDLINE search terms	4
Appendix 3: Table of indicative SDG-related outcomes, and elements on the pathway between a change in eye health and an SDG.	7
Appendix 4: Copy of Google Form used for data extraction	8
Appendix 5: Table of studies excluded post-hoc due to small sample sizes (N<100)	10
Appendix 6: Table of included studies (N=29)	12
Appendix 7: Summary of evidence linking specific services or interventions to improve eye health and specific SDGs.	17
References	18

Appendix 1: Preferred Reporting Items for Systematic reviews and Meta-Analyses extension for Scoping Reviews (PRISMA-ScR) Checklist

SECTION	ITEM	PRISMA-ScR CHECKLIST ITEM	REPORTED ON PAGE #
**TITLE**			
Title	1	Identify the report as a scoping review.	1
**ABSTRACT**			
Structured summary	2	Provide a structured summary that includes (as applicable): background, objectives, eligibility criteria, sources of evidence, charting methods, results, and conclusions that relate to the review questions and objectives.	1
**INTRODUCTION**			
Rationale	3	Describe the rationale for the review in the context of what is already known. Explain why the review questions/objectives lend themselves to a scoping review approach.	1
Objectives	4	Provide an explicit statement of the questions and objectives being addressed with reference to their key elements (e.g., population or participants, concepts, and context) or other relevant key elements used to conceptualize the review questions and/or objectives.	1
**METHODS**			
Protocol and registration	5	Indicate whether a review protocol exists; state if and where it can be accessed (e.g., a Web address); and if available, provide registration information, including the registration number.	1
Eligibility criteria	6	Specify characteristics of the sources of evidence used as eligibility criteria (e.g., years considered, language, and publication status), and provide a rationale.	1-2
Information sources*	7	Describe all information sources in the search (e.g., databases with dates of coverage and contact with authors to identify additional sources), as well as the date the most recent search was executed.	1
Search	8	Present the full electronic search strategy for at least 1 database, including any limits used, such that it could be repeated.	Supplementary appendix 2
Selection of sources of evidence†	9	State the process for selecting sources of evidence (i.e., screening and eligibility) included in the scoping review.	2
Data charting process‡	10	Describe the methods of charting data from the included sources of evidence (e.g., calibrated forms or forms that have been tested by the team before their use, and whether data charting was done independently or in duplicate) and any processes for obtaining and confirming data from investigators.	2-3
Data items	11	List and define all variables for which data were sought and any assumptions and simplifications made.	2-3
Critical appraisal of individual sources of evidence§	12	If done, provide a rationale for conducting a critical appraisal of included sources of evidence; describe the methods used and how this information was used in any data synthesis (if appropriate).	N/A
Synthesis of results	13	Describe the methods of handling and summarizing the data that were charted.	3
**RESULTS**			
Selection of sources of evidence	14	Give numbers of sources of evidence screened, assessed for eligibility, and included in the review, with reasons for exclusions at each stage, ideally using a flow diagram.	3; Figure 1
Characteristics of sources of evidence	15	For each source of evidence, present characteristics for which data were charted and provide the citations.	3; Table 1
Critical appraisal within sources of evidence	16	If done, present data on critical appraisal of included sources of evidence (see item 12).	N/A
Results of individual sources of evidence	17	For each included source of evidence, present the relevant data that were charted that relate to the review questions and objectives.	3-4; Table 2; supplementary appendix 6
Synthesis of results	18	Summarize and/or present the charting results as they relate to the review questions and objectives.	3-4; Figures 2 & 3
**DISCUSSION**			
Summary of evidence	19	Summarize the main results (including an overview of concepts, themes, and types of evidence available), link to the review questions and objectives, and consider the relevance to key groups.	4-8
Limitations	20	Discuss the limitations of the scoping review process.	8
Conclusions	21	Provide a general interpretation of the results with respect to the review questions and objectives, as well as potential implications and/or next steps.	9
**FUNDING**			
Funding	22	Describe sources of funding for the included sources of evidence, as well as sources of funding for the scoping review. Describe the role of the funders of the scoping review.	9

JBI = Joanna Briggs Institute; PRISMA-ScR = Preferred Reporting Items for Systematic reviews and Meta-Analyses extension for Scoping Reviews.

* Where *sources of evidence* (see second footnote) are compiled from, such as bibliographic databases, social media platforms, and Web sites.
† A more inclusive/heterogeneous term used to account for the different types of evidence or data sources (e.g., quantitative and/or qualitative research, expert opinion, and policy documents) that may be eligible in a scoping review as opposed to only studies. This is not to be confused with *information sources* (see first footnote).
‡ The frameworks by Arksey and O'Malley (6) and Levac and colleagues (7) and the JBI guidance (4, 5) refer to the process of data extraction in a scoping review as data charting.
§ The process of systematically examining research evidence to assess its validity, results, and relevance before using it to inform a decision. This term is used for items 12 and 19 instead of "risk of bias" (which is more applicable to systematic reviews of interventions) to include and acknowledge the various sources of evidence that may be used in a scoping review (e.g., quantitative and/or qualitative research, expert opinion, and policy document).

From: Tricco AC, Lillie E, Zarin W, O'Brien KK, Colquhoun H, Levac D, et al. PRISMA Extension for Scoping Reviews (PRISMA-ScR): Checklist and Explanation. Ann Intern Med. ;169:467–473. doi: 10.7326/M18-0850

Appendix 2: MEDLINE search terms

1. exp Ophthalmology/
2. exp Eye Diseases/
3. (trachoma$ or tracoma$ or trichiasis).tw.
4. (cataract$ or phaco$ or phako$).tw.
5. ((diabet$ or proliferat$) adj3 retinopath$).tw.
6. (amblyop$ or strabismus).tw.
7. exp Vision Tests/
8. Optometry/
9. (myopia or myopic or hyperop$ or hypermetrop$ or anisometrop$ or ammetrop$ or astigmati$ or presbyop$).tw.
10. (refractive adj1 error$).tw.
11. Eyeglasses/
12. (spectacle or spectacles or glasses).tw.
13. (eyeglasses or eye glasses).tw.
14. ((eye$ or vision or retina$ or ophthalm$ or retinopathy) adj2 exam$).tw.
15. ((eye$ or vision or retinopathy or ophthalm$) adj2 assess$).tw.
16. ((eye$ or vision or retina$ or ophthalm$ or retinopathy) adj2 test$).tw.
17. (eye$ adj2 (care or health or service$)).tw.
18. onchocerciasis.tw.
19. (river adj1 blindness).tw.
20. (retinopath$ adj2 prematur$).tw.
21. or/1-20
22. Trachoma/
23. (trachoma$ or tracoma$ or trichiasis).tw.
24. exp Cataract/
25. exp cataract Extraction/
26. cataract$.tw.
27. or/22-26
28. exp poverty/
29. poverty.tw.
30. poor people.tw.
31. (social adj2 protection).tw.
32. household expenditure.tw.
33. head of household.tw.
34. per capita expenditure.tw.
35. asset ownership.tw.
36. self-rated wealth.tw.
37. or/28-36
38. Malnutrition/
39. Malnutrition.tw.
40. (malnourish$ or undernourish$).tw.
41. (nutrition$ adj2 deficienc$).tw.
42. Vitamin A Deficiency/
43. Vitamin A deficien$.tw.
44. or/38-43
45. Education/
46. "Education of Visually Disabled"/
47. "Education of Intellectually Disabled"/
48. Remedial Teaching/
49. (school adj3 (performance or attain$ or achieve$ or attend$)).tw.
50. (educat$ adj2 (need$ or require$ or strateg$ or outcome$ or develop$)).tw.
51. (reading adj2 (attain$ or develop$)).tw.
52. (literacy or illiteracy or illiterate or numeracy).tw.
53. or/45-52
54. Women's Health/
55. Women's Health Services/
56. exp "sexual and gender minorities"/
57. (transgender or transsexual$).tw.

58. (gender adj1 identit$).tw.
59. (women$ adj1 health$).tw.
60. or/54-59
61. exp environmental health/
62. exp hygiene/
63. exp water supply/
64. ((wash$ or clean$ or hygien$) adj2 face$).tw.
65. ((wash$ or clean$ or hygien$) adj2 facial$).tw.
66. Insect Control/
67. exp insecticides/
68. or/61-67
69. Renewable Energy/
70. Energy-Generating Resources/
71. Power plants/
72. or/69-71
73. exp Employment/
74. Efficiency/
75. Efficiency, Organizational/
76. Sickness impact profile/
77. Occupational Diseases/
78. Absenteeism/
79. Presenteeism/
80. productivity.tw.
81. (product$ adj2 employ$).tw.
82. or/73-81
83. Organizational Innovation/
84. Sustainable Development/
85. or/83-84
86. Social Justice/
87. ((social$ or econom$ or politic$) adj3 inclu$).tw.
88. Healthcare Disparities/
89. Health Status Disparities/
90. (inequal$ or inequit$).tw.
91. equal opportun$.tw.
92. or/86-91
93. exp Motor Vehicles/
94. exp Automobile Driving/
95. exp Accidents, Traffic/
96. (road adj2 safe$).tw.
97. ((accident$ or crash$ or collision$) adj2 (road or car or vehicle$ or motor$ or traffic)).tw.
98. or/93-97
99. exp "Conservation of Natural Resources"/
100. exp Climate/
101. exp climate change/
102. (climate adj2 chang$).tw.
103. or/100-102
104. 37 or 44 or 53 or 60 or 72 or 82 or 85 or 92 or 98 or 99
105. 21 and 104
106. 68 or 103
107. 27 and 106
108. 105 or 107
109. exp animals/
110. exp humans/
111. 109 not (109 and 110)
112. 108 not 111
113. (animal$ or rabbit$ or rat or rats or mouse or mice or chicken$ or dog or dogs or cat or cats or feline or pig$ or monke$).tw.
114. 112 not 113
115. exp case reports/
116. (case$ adj3 (report$ or series)).tw.

117. 115 or 116
118. 114 not 117
119. (immunoblot$ or electron or mRNA or mitochondria$ or proteomic$ or metabolomic$ or allele or extracellular).tw.
120. (in vivo or in-vivo or in vitro or in-vitro).tw.
121. Urinary Tract Infections/
122. Gonorrhea/
123. Sexually Transmitted Diseases/
124. vagina/
125. pelvic inflammatory disease$.tw.
126. (gonorrhea$ or gonorrhoea$).tw.
127. (sexual$ adj3 transmit$).tw.
128. HIV.tw.
129. (ureth$ or urinary or genital$).tw.
130. (vagina$ or testes or testic$).tw.
131. visual analog$ scale$.tw.
132. (dental or oral$).ti.
133. or/119-132
134. 118 not 133

Appendix 3: Table of indicative SDG-related outcomes, and elements on the pathway between a change in eye health and an SDG.

**SDG**	**SDG-related outcomes**	**Elements on the pathway**
SDG 1: No poverty	• Poverty (all dimensions)<br>• Access to economic resources and microfinance initiatives	• Productivity<br>• Household per capita expenditure<br>• Social protection schemes<br>• Financial burden on, and sustainability of, the health system<br>• Efficient and effective use of resources<br>• Universal health coverage
SDG 2: Zero Hunger	• Nutrition<br>• Food security	• Vulnerability to malnutrition at the individual level<br>• Agricultural production
SDG 4: Quality education	• Educational outcomes such as literacy and numeracy rates<br>• Skills attainment for future employment<br>• Disparities in access to education / inclusive education	• School enrolment, attendance and retention
SDG 5: Gender equality	• Gender discrimination<br>• Violence against women and girls	• Gender equity in access to eye care services<br>• Empowerment of women and girls
SDG 6: Clean water and sanitation	• Access to safe, affordable drinking water<br>• Access to adequate sanitation and hygiene	• Strengthening of infrastructure for improved water, sanitation and hygiene
SDG 7: Affordable and clean energy	• Renewable energy<br>• Energy efficiency	
SDG 8: Decent work and economic growth	• Per capita economic growth<br>• Employment and decent work opportunities	• Worker productivity<br>• Presenteeism<br>• Absenteeism<br>• Retirement age
SDG 9: Industry, innovation and infrastructure	• Quality infrastructure<br>• Sustainable industrialisation<br>• Expansion of technology and innovation	
SDG 10: Reduced inequalities	• Social inclusion of all<br>• Economic inclusion of all<br>• Political inclusion of all	• Empowerment of vulnerable groups
SDG 11: Sustainable cities and communities	• Access to safe and affordable transport<br>• Access to safe and affordable housing<br>• Environmental impact of cities	• Road safety<br>• Environmental impact of the healthcare system, e.g. waste management
SDG 12: Responsible consumption and production		• Environmental impact of the healthcare system, e.g. waste management<br>• Recycling of eye care consumables
SDG 13: Climate action		
SDG 14: Life below water		
SDG 15: Life on land		
SDG 16: Peace, justice and strong institutions	• Levels of violence<br>• Levels of corruption	
SDG 17: Partnership	• Coalition-building partnerships	

Appendix 4: Copy of Google Form used for data extraction

SDGs and Eye Health
*Required

1. Email address *

2. Form filled by: *

Publication Characteristics

3. First author (Surname)

4. Year published

5. Countries of study
If no information available, please input 'NA'.

6. Year(s) performed.
If no information is available on 'year(s) performed', then please input 'year(s) of data included for analysis'. If no information available for either, please input 'NA'.

7. Source of funding
If no information available, please input 'NA'.

8. Please copy and paste the Funding/Financial Disclosure statement here.
If the paper does not have a statement, please input 'NA'.

Study details

9. Intervention(s)

10. SDG-related outcome(s) reported in the paper
(Please write the SDG-related outcome here -- there is no need to specify the exact SDG here, as there is a later question asking you to tick the relevant SDG.)

11. Does the paper report an effect estimate? (e.g. odds ratio, relative risk, etc)
(Not including p-values -- see next question for p-values)

12. Does the paper report p-values or confidence intervals?

SDG-related characteristics

The following logic model is intended as a guide and is not restrictive. If there are additional pathways to the achievement of an SDG that are not mapped out on this model, please still tick the relevant SDG as we will adapt the model based on the data extraction results.

Figure 1: Logic model outlining examples of pathways by which improved eye health may contribute to the achievement of the sustainable development goals.

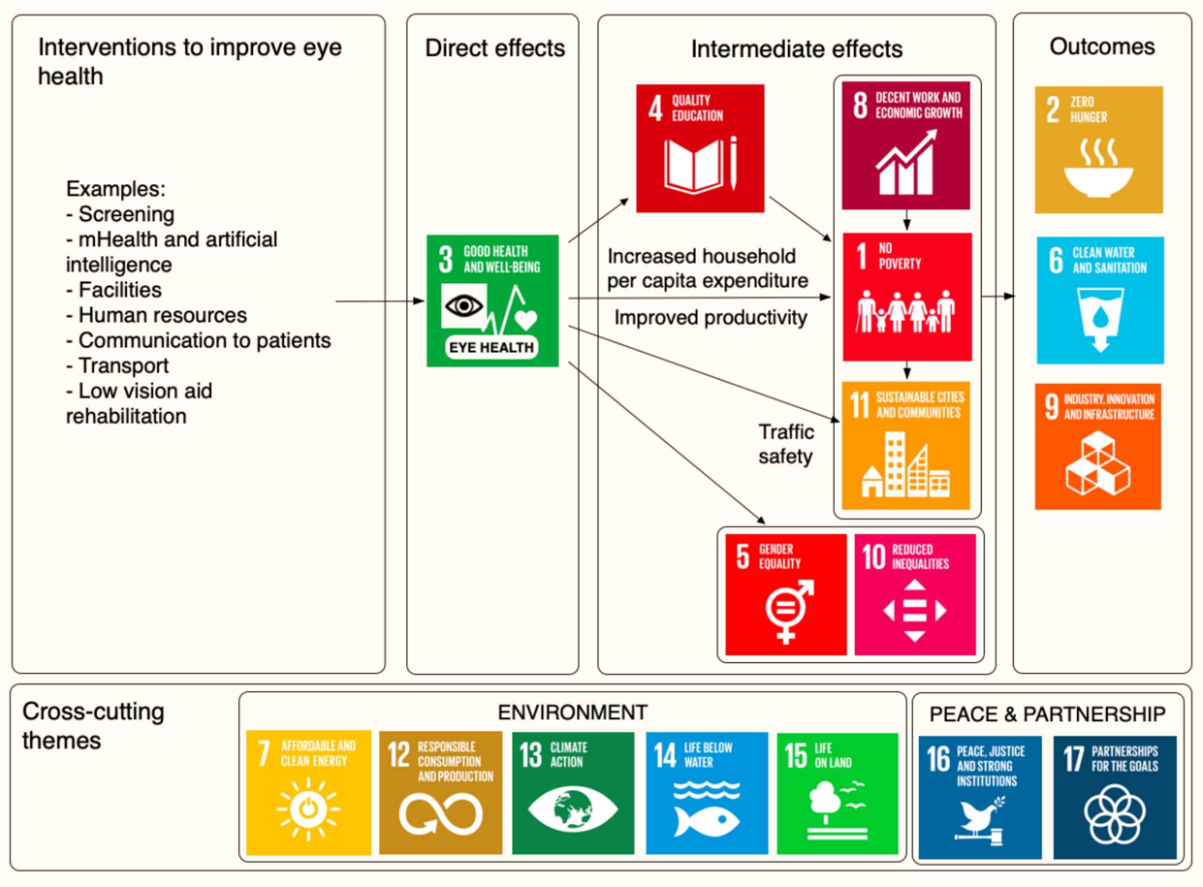

13. Identify relevant SDG(s) - *Tick all that apply.*

SDG 1: End Poverty
SDG 2: Zero Hunger
SDG 4: Quality Education
SDG 5: Gender Equality
SDG 6: Clean Water and Sanitation
SDG 7: Affordable and Clean Energy
SDG 8: Decent Work and Economic Growth
SDG 9: Industry, Innovation, and Infrastructure
SDG 10: Reduced Inequalities
SDG 11: Sustainable Cities and Communities
SDG 12: Responsible Consumption and Production
SDG 13: Climate Action
SDG 14: Life Below Water
SDG 15: Life On Land
SDG 16: Peace, Justice, and Strong Institutions
SDG 17: Partnerships for the Goals

14. Does the paper address any cross-cutting themes of environmental sustainability?

15. Does the paper address any cross-cutting themes of peace and partnership?

Appendix 5: Table of studies excluded post-hoc due to small sample sizes (N<100)

Outcomes	Study design	Study aims relevant to the linked SDG	No. of participants	Study findings
Financial self-sufficiency	Retrospective cohort study	To compare the financial self-sufficiency of congenitally legally blind adults who learned to read braille versus print as their original reading medium in the USA.[1]	74	Congenitally legally blind adults who learned Braille were more financially self-sufficient, than did those who learned to read using print (p=0·028).
Workplace productivity	Prospective cohort studies	To examine the association between the astigmatic refractive corrections of computers users and error rates in the completion of computer tasks.[2]	39	Correction of astigmatism reduced errors in computer tasks (no statistical test).
		To evaluate the association of workplace lighting environment of postal workers in Sweden and mail sorting time.[3]	25	After the provision of improved workplace lighting for postal workers, there was an improvement in the time to sort 150 E5 letters of 0·1 seconds per letter for the entire group.
Employment rates	Retrospective cohort studies	To compare the employment rates of congenitally legally blind adults who learned to read braille versus print as their original reading medium in the USA.[1]	74	Congenitally legally blind adults who learned Braille had lower unemployment rates than did those who learned to read using print (p<0·0148).
		To compare the employment rate of people with vision impairment who received 'occupational training' with people who did not receive 'occupational training' in Japan[4]	45	The national employment rate in Japan in people with vision impairment was 27·4% in 1996. In comparison, the employment rate in the intervention group who received 'occupational training' between 1996 and 2001 was 73·3%.

Outcomes	Study design	Study aims relevant to the linked SDG	No. of participants	Study findings
Academic test scores	Prospective cohort studies	To compare a conventional Braille book program for teaching blind students in Germany to spell versus an electronic teaching system called 'BRAILLOPHON'.[5]	68	Test scores were higher in the group who completed the 'BRAILLOPHON' teaching program versus the conventional Braille book program for teaching blind students in Germany to spell (p<0·05).
		To compare the reading and spelling abilities of children with vision and/or perceptual disorders in the USA who had perceptual therapy and visual training compared to controls who did adaptive physical education, art or science instead.[6]	36	Children with vision and/or perceptual disorders who had perceptual therapy and visual training had improved scores in reading performance and spelling tests compared to controls.
Reading/word identification	Randomised controlled trial	To compare the effect of Lawson orthoptic and adapted Phono-Graphix intervention programs versus a control group (parental literacy support only) on educational outcomes in children with low literacy and poor stereoacuity in Australia.[7]	88	At the 36-week follow-up appointment, the Lawson orthoptic and adapted Phono-Graphix improved word identification but not any other educational outcomes.
	Prospective cohort study	To assess how bifocals change the development of literacy skills in children with Down syndrome presenting with inadequate accommodation.[8]	14	Bifocals improved word identification test scores (p=0·008) in children with Down syndrome presenting with inadequate accommodation.
	Retrospective cohort study	To compare the amount of time spent reading of congenitally legally blind adults who learned to read braille versus print as their original reading medium in the USA.[1]	74	A greater proportion of congenitally legally blind adults who learned Braille spent more than 21 hours a week reading than did those who learned to read using print, in a study in the US (p<0·017).

	Effective learning	Prospective cohort studies	To assess the impact of early detection of vision impairment and remediation on learning in children in the USA.[9]	25	Early detection of vision impairment and remediation increased the potential for more effective learning in 25 children.
	Developmental Visual-Motor Integration	Prospective cohort study [10]	To compare developmental Visual-Motor Integration test results in pre-schoolers in the USA who wore spectacles for 6 weeks versus emmtropic controls.	70	Pre-schoolers with ametropia who wore spectacles for 6 weeks improved on the developmental Visual-Motor Integration test compared with emmetropic control subjects (p=0·02).

11 SUSTAINABLE CITIES AND COMMUNITIES	**Outcomes**	**Study design(s)**	**Study aims relevant to the linked SDG**	**No. of participants**	**Study findings**
	Driving performance	Exposure cross-over study	To investigate the effect of correcting astigmatism using toric contact lenses in adults with astigmatism on night-time driving performance.[11]	10	Toric lens provision to correct astigmatism improved night-time driving performance among adults with low-to-moderate bilateral astigmatism (p<0·05).
		Prospective cohort studies	To investigate whether cataract surgery improves driving performance.[12]	47	At baseline, driving performance (evaluated by a scoring system on a closed-circuit road assessment) of people with bilateral cataracts was significantly worse than people with normal vision (p=0·01). After cataract surgery, the driving performance of the cataract group improved significantly (p=0·001).
			To compare driving performance of visually impaired drivers using bioptic telescopes versus age-matched controls in the USA.[13]	46	Drivers with central vision loss who were licensed to drive through a bioptic driving program displayed proficient on-road driving skills. 96% (22/23) of licensed bioptic drivers were rated as safe to drive by the evaluators and 100% (23/23) of controls were rated as safe to drive.

Appendix 6: Table of included studies (N=29)

Evidence (including randomised controlled trial evidence) shows that common and inexpensive eye health interventions such as spectacles and cataract surgery can increase workplace productivity, household per capita expenditure, household income, employment rates, and national economic productivity. Reduction of poverty is a major factor in achieving zero hunger. Evidence indicating how eye health might impact on SDGs 1, 2 and 8 includes:

**Outcomes**	**Study design**	**Study aims relevant to the linked SDG**	**No. of participants**	**Study findings**
Workplace relative productivity	Randomised controlled trial	To assess whether providing free glasses to tea workers with presbyopia in India improves work productivity.[14]	751	Provision of free spectacles to tea workers with presbyopia improved workplace relative productivity by 21·7% (picking an extra 5·25Kg of tea per day; 95%CI 4·50-5·99; 21·7% relative productivity increase; effect size 1·01 [95% CI 0·86–1·16]; p<0·0001).
Household per capita expenditure	Prospective cohort studies	To assess the six-year impact of cataract surgery on economic poverty in Bangladesh and the Philippines.[15]	898	At baseline, people with visual impairment from cataract were poorer compared to controls without vision impairment in terms of self-reported household per capita expenditure (Philippines $22 vs $29 per person per month, p=0·04; Bangladesh $15 vs $27, p=0·02). Six years after cataract surgery, household per capita expenditure increased in people with operated cataract such that levels became similar to that of the control group (Philippines $39 vs $37 p=0.85, Bangladesh $23 vs $24 p=0·85).
		To assess whether there is a reduction in poverty after cataract surgery among visually impaired cases in Kenya, the Philippines, and Bangladesh.[16]	902	At baseline, people with visual impairment from cataract were poorer compared to controls without vision impairment in terms of self-reported household per capita expenditure (Kenya: $22 versus £35 p=0·02, Bangladesh: $16 vs $24 p=0·004, Philippines: $24 vs 32 p=0·0007). One year after cataract surgery, household per capita expenditure increased in people with operated cataract such that levels became similar to that of the control group (Kenya: $30 versus £36 p=0·49, Bangladesh: $23 vs $23 p=0·20, Philippines: $45 vs $36 p=0·68) and average household per capita expenditure had increased among people with operated cataract by $8 in Kenya (36%, p=0·07), $7 in Bangladesh (44%, p<0·0001) and $21 in the Philippines (88%, p<0·0001).
Household income	Prospective cohort studies	To assess whether cataract surgery provided to impoverished rural communities in India improves poverty.[17]	294	One year after provision of free high-quality cataract surgery to marginalized rural communities in India, more participants were engaged in income generating activities compared to baseline (44·7% to 77·7%; p<0·001), and the proportion of households with a monthly income <1000 Rps. decreased from 50·5% to 20·5% (p=0·05).
		To measure the change in economic circumstances after cataract surgery in Vietnam.[18]	480	Cataract surgery in Vietnam improved household economic circumstances from baseline to 12-month follow-up:<br>- mean increase in paid work participation per month: 44·5 hours, P<0·0001;<br>- decrease in proportion with hardship: -17%, P < 0·0001;<br>- decrease in proportion with catastrophic health expenditure: -7%, P=0·02.
		To assess the impact of cataract surgery in Ethiopia on household incomes.[19]	1,234	Among people who received cataract surgery in Ethiopia, 36% self-reported ‘a little increase’ in household income/production at 1-year follow-up compared to baseline, 5% self-reported that their household income/production increased ‘a lot’, 59% reported no change in household income/production, and no-one reported a reduction in household income/production.
	Retrospective cohort study	To investigate the relationship between type of school attended	270	*In people who became legally blind by the age of 6 years in the USA, those who attended specialised schools for people with vision impairment, had a lower salary (mean gross salary $171·03) compared to those who attended public schools (mean*

		and gross salary of legally blind adults in the USA.[20]		*gross salary $205·92), although this may have been confounded by other determinants.*
Employment rates	Retrospective cohort study	To assess the impact of vocational rehabilitation services on paid employment outcomes for people with vision impairment in the USA.[21]	3,610	Some vocational rehabilitation services for people with vision impairment in the USA were positively associated with paid employment. For example, 'training and support' services were associated with increased odds of obtaining paid employment (OR 1·10, p=0·001, two-level hierarchical generalized linear model).
National economic productivity/gain	Cost-effectiveness studies	To compare cost utility data for cataract surgery in the USA in 2000 and 2012.[22]	NA	The 13-year financial return on investment (societal perspective) for first-eye cataract surgery was US$121,198: a 4567% gain. There was a net 13-year US$123·4-billion return on investment from a 1-year cohort of cataract surgery patients which included an increase in United States national productivity of US$25·4 billion.
	Cost-evaluation studies	To assess the economic productivity of people who had trichiasis surgery in The Gambia.[23]	NA	The total lifetime productivity gain is estimated at US$89 for individuals who had trichiasis surgery in The Gambia.
	Cost-of illness studies	To estimate the cost of illness of untreated trichiasis and compare this with the actual cost of providing trichiasis surgery in The Gambia.[24]	NA	The estimated economic productivity lost due to trachomatous vision impairment in The Gambia was more than ten times the total cost of providing local surgery, even with a series of assumptions biasing the cost of illness downward.
		To compare the costs and benefits, in the UK, of an outreach clinic for cataract day surgery in a small community hospital versus cataract day surgery in a large district general hospital.[25]	399	The net benefit to patients of relocating cataract surgery to a community outreach service compared to a hospital service in the UK was estimated as £39,000 per annum.

4 QUALITY EDUCATION

Evidence (including randomised controlled trial evidence) shows that improved vision for children improves educational performance. Evidence indicating that eye health can help achieve SDG 4 includes:

**Outcomes**	**Study design**	**Study aims relevant to the linked SDG**	**No. of participants**	**Study findings**
Academic test scores	Randomised controlled trials	To estimate the size of the impact of spectacles on academic test scores in rural Western China.[26]	18,902	In rural Western China, a programme to provide spectacles to children with poor vision for one academic year increased their average test scores by 0·16 standard deviations, equivalent to 0·3 additional years of schooling, compared to those who were not in the programme.
		To quantify the impact of screening along with free eye exams and free spectacles in the USA on student test scores.[27]	4,968	Providing screening along with free eye exams and free spectacles in the USA increased the probability of passing the Florida Comprehensive Achievement Tests in reading and maths by approximately 2·0 percentage points, although only the reading test reaches statistical significance.
		To quantify the impact of spectacles on class failure, literacy scores, and maths scores, in Gansu province, China.[28]	19,185	Vision correction with spectacles reduced the odds of failing a class by 44% (p<0·01) and improved literacy scores (treatment effect of 0·34 standard deviations) and maths scores (treatment effect of 0·26 standard deviations), in Gansu province, China.

		To assess the effect of provision of free spectacles on academic performance in Chinese children with myopia.[29]	3,177	Chinese children with myopia who were not provided with free spectacles (control group) had an increase in test score of 0·07 standard deviations (95%CI −0·0002 to 0·14) between the start and end of the school year. In contrast, children who were provided with free spectacles had an increase in test score of 0·16 standard deviations (95% CI 0·07 to 0·24) between the start and end of the school year. When compared with the control group, the provision of free spectacles to children with myopia improved children's performance in maths tests: the adjusted difference in test score was 0·11 standard deviations (95% CI 0·01-0·21), equivalent to approximately half a semester of additional learning.
		To study the effect of early versus late referral for refraction on academic performance in Chinese children.[30]	1,200	In a group of children in China who received late referral for refraction and free spectacles, mean mathematics score at the end of the study was -0·16 (SD 0·97). In contrast, in children who received early referral for refraction and free spectacles, mean mathematics score at the end of the study was 0·14 (SD 1·01). The adjusted effect on mathematics test scores was 0·25 standard deviations (95% CI, 0·01-0·48; 1-sided *P* = 0·04), equivalent to half a semester of additional learning, when comparing the group who received early referral for refraction and free spectacles, versus the group who received late referral.
	Prospective cohort studies	To assess the impact of spectacles on standardized testing scores in children in the USA.[31]	4,523	In the USA, when spectacles were prescribed and worn by Asian children, higher scores were achieved in the Pennsylvania System of School Assessment Reading examination (OR = 2·53, p = 0·004). This trend was also observed in black and Hispanic children without reaching statistical significance (OR=1·70, p=0·061; OR=2·67, p=0·067, respectively). A similar benefit of spectacles was found in children of other racial groups, but this did not reach statistical significance.
		To investigate the effect of refractive error correction on achievement scores in primary school children in Kerala, India.[32]	185	Primary school children with refractive error in Kerala, India, had a total achievement score of 20·5 before correction of their refractive error, but after correction of their refractive error, total achievement score improves to 27.1 (p<0·01).
Reading ability	Prospective cohort study	To investigate the impact of adherent versus non-adherent spectacle wear on letter identification scores.[33]	944	Children in Bradford, UK, who were adherent to spectacle wear consistently demonstrated higher letter identification scores compared with the non-adherent group, with the greatest effect size in year 3 (0·11, Cohen's d).
	Retrospective cohort study	To investigate the braille reading ability of legally blind adults in the USA who attended specialised schools for people with vision impairment compared to public schools.[20]	270	A greater proportion of legally blind adults who attended specialised schools in the USA could read braille compared to legally blind adults who attended public schools (72·2% vs 19·7%, p<0·001).

There is some evidence showing that interventions to improve eye health drive a reduction in inequity, but also one study showing that eye health services increased inequity. There is scope for further research in this area. Evidence indicating how eye health impacts on SDG 5 & 10 includes:

**Outcomes**	**Study design**	**Study aims relevant to the linked SDG**	**No. of participants**	**Study findings**
Gender equality	Pair of cross-sectional surveys	To compare willingness to pay at least 500 RMB for cataract surgery in China in men vs women, after the introduction of a programme offering free cataract screening and	303	Two cross-sectional surveys in 2001 and 2006, conducted following the introduction of a programme in 2001 that offered free cataract screening and low-cost high-quality cataract surgery in China, showed that willingness to pay at least 500 RMB for cataract surgery increased from 67% to 88% among men (p<0·01) and from 50% to 91% among women (p<0·01).

		low-cost high-quality cataract surgery.[34]		
	Systematic review and meta-analysis	To identify evidence regarding interventions to increase gender equity in eye care.[35]	NA	Included articles in this systematic review were of low to very low quality. Six studies examined interventions to train rural community volunteers to assist people with unmet eye care needs in low-middle income countries, and the interventions were associated with reduced gender inequality in all-cause blindness, clinic attendance, cataract surgery coverage, and trachoma treatment coverage. Interventions to improve paediatric cataract surgery follow-up in Nepal and Tanzania found reduced gender inequality in 10-week follow-up rates.
Equality in utilisation of services, by socioeconomic and educational status	Series of annual cross-sectional surveys	To assess differences in the way people across different socio-economic groups responded to the introduction of free eye examinations in Scotland.[36]	Number of participants not stated, but uses two large datasets: British Household Panel Survey and records from a private optician company.	*Introduction of free eye examinations in Scotland in 2006 widened inequalities in utilisation of eye care services when comparing high vs low income groups (p<0·001), and high vs low education groups (p<0·001).*
	Prospective cohort study	To assess whether there is a reduction in poverty after cataract surgery among visually impaired cases in Kenya, the Philippines, and Bangladesh.[16]	902	Cataract surgery can contribute to poverty alleviation, particularly among the most vulnerable members of society. At baseline, operated cataract cases had significantly lower per capita expenditure compared to controls (Kenya: p=0·02; Bangladesh: p=0·004; the Philippines: p=0·0007). At 1-year follow up, per capita expenditure was no longer significantly lower than among controls (p≥0·2 for all 3 countries).

Treatment of conditions such as cataract can lead to reduced risk of motor vehicle crashes and safer streets (Target 11.2). Evidence indicating that eye health can help achieve SDG 11 includes:

**Outcomes**	**Study design**	**Study aims relevant to linked SDG**	**No. of participants**	**Study findings**
Driving performance	Meta-analysis	To assess the effects of cataract surgery in improving vision and driving performance while reducing driving-related difficulties.[37]	1,642	A meta-analysis showed that the risk of driving-related difficulties was reduced by 88% following cataract surgery (pooled OR 0·12, 95% CI 0·10 – 0·16).
Motor vehicle crashes	Prospective cohort study	To assess the impact of cataract surgery on crash risk in older adults in the USA.[38]	277	Cataract surgery halved the rate of car crash involvement compared to patients with cataract who did not have surgery in the USA (rate ratio 0·47, 95%CI 0·23-0·94).
	Retrospective cohort studies	To evaluate the association between first- and second-eye cataract surgery and crash risk for older drivers in Western Australia.[39]	2,849	Motor vehicle crash rates were 21·6 crashes per 100,000 km travelled before first eye cataract surgery; 7·6 crashes per 100,000 km travelled between first and second eye cataract surgery; and 17·1 crashes per 100,000 km travelled after second eye surgery. First eye cataract surgery was associated with a 61% reduction in motor vehicle crashes (p<0·001) and second eye cataract surgery was associated with a 23% reduction in motor vehicle crashes (p<0·001), compared to the year before first eye cataract surgery.
		To evaluate the effectiveness of first eye cataract surgery on drivers aged ≥60 years in Western Australia.[40]	27,827	There was a significant reduction of 12·7% (p=0·034) in all police reported crashes one year after cataract surgery compared to one year before cataract surgery.

				To determine gender- differences in the effectiveness of first eye cataract surgery in reducing motor vehicle crashes in older drivers in Western Australia.[41]	1,715	Significant reduction of 15·3% (P=0·040) in all police reported crash frequency for men one year after cataract surgery versus before surgery. No significant change in crash frequency for women one year after cataract surgery versus before surgery.
			Exposure cross-over study	To assess whether cataract surgery is associated with reduced motor vehicle crashes where the patient was the driver.[42]	559,546	In a Canadian study, cataract surgery was associated with 0·22 fewer crashes per 1000 patient-years following cataract surgery (odds ratio 0·91, 95% CI 0·84-0·97, p=0·004).

Findings that resulted in a negative outcome (i.e. an intervention resulting in a worsening of an SDG-related outcome) are highlighted in *italics*. Abbreviations: CI, confidence interval; NA, not applicable; OR, odds ratio.

Appendix 7: Summary of evidence linking specific services or interventions to improve eye health and specific SDGs. Numbers inside boxes indicate number of studies. Green indicates direct positive benefit (N=27) and grey indicates a negative relationship (N=2). Intensity of colour represents the number of studies reporting the relationship.

**Service/intervention**	**Poverty-related SDGs 1, 2 and 8**	**Education SDG 4**	**Equality SDGs 5 and 10**	**Sustainable cities SDG 11**
Cataract surgery	6		1	6
Trichiasis surgery	2			
Spectacles	1	7		
Vocational rehabilitation services	1			
Training rural community eye health volunteers			1	
Specialised schools for visually impaired children	1	1		
Community outreach	1			
Free/low-cost eye services			1<br>1	
Early referral to an eye health centre		1		

References

1. Ryles R. The impact of braille reading skills on employment, income, education, and reading habits. *Journal of Visual Impairment and Blindness* 1996; **90**(3): 219-26.
2. Daum KM, Clore KA, Simms SS, et al. Productivity associated with visual status of computer users. *Optometry* 2004; **75**(1): 33-47.
3. Hemphala H, Eklund J. A visual ergonomics intervention in mail sorting facilities: effects on eyes, muscles and productivity. *Appl Ergon* 2012; **43**(1): 217-29.
4. Kikuiri A, Tabuchi A. Occupational status of the visually disabled in Japan. *Folia Ophthalmologica Japonica* 2004; **55**(7): 566-70.
5. Nater P. An electronic teaching device for blind students. *Journal of Visual Impairment and Blindness* 1982; **76**(7): 274-8.
6. Seiderman AS. Optometric vision therapy -- results of a demonstration project with a learning disabled population. *J Am Optom Assoc* 1980; **51**(5): 489-93.
7. Ponsonby AL, Williamson E, Smith K, et al. Children with low literacy and poor stereoacuity: an evaluation of complex interventions in a community-based randomized trial. *Ophthalmic Epidemiol* 2009; **16**(5): 311-21.
8. Nandakumar K, Evans MA, Briand K, Leat SJ. Bifocals in Down syndrome study (BiDS): analysis of video recorded sessions of literacy and visual perceptual skills. *Clin Exp Optom* 2011; **94**(6): 575-85.
9. Krumholtz I. Results from a pediatric vision screening and its ability to predict academic performance. *Optometry* 2000; **71**(7): 426-30.
10. Roch-Levecq AC, Brody BL, Thomas RG, Brown SI. Ametropia, preschoolers' cognitive abilities, and effects of spectacle correction. *Arch Ophthalmol* 2008; **126**(2): 252-8; quiz 161.
11. Black AA, Wood JM, Colorado LH, Collins MJ. The impact of uncorrected astigmatism on night driving performance. *Ophthalmic Physiol Opt* 2019; **39**(5): 350-7.
12. Wood JM, Carberry TP. Bilateral cataract surgery and driving performance. *Br J Ophthalmol* 2006; **90**(10): 1277-80.
13. Wood JM, McGwin G, Jr., Elgin J, Searcey K, Owsley C. Characteristics of on-road driving performance of persons with central vision loss who use bioptic telescopes. *Invest Ophthalmol Vis Sci* 2013; **54**(5): 3790-7.
14. Reddy PA, Congdon N, MacKenzie G, et al. Effect of providing near glasses on productivity among rural Indian tea workers with presbyopia (PROSPER): a randomised trial. *Lancet Glob Health* 2018; **6**(9): e1019-e27.
15. Danquah L, Kuper H, Eusebio C, et al. The long term impact of cataract surgery on quality of life, activities and poverty: results from a six year longitudinal study in Bangladesh and the Philippines. *PLoS One* 2014; **9**(4): e94140.
16. Kuper H, Polack S, Mathenge W, et al. Does cataract surgery alleviate poverty? Evidence from a multi-centre intervention study conducted in Kenya, the Philippines and Bangladesh. *PLoS One* 2010; **5**(11): e15431.
17. Finger RP, Kupitz DG, Fenwick E, et al. The impact of successful cataract surgery on quality of life, household income and social status in South India. *PLoS One* 2012; **7**(8): e44268.
18. Essue BM, Li Q, Hackett ML, et al. A multicenter prospective cohort study of quality of life and economic outcomes after cataract surgery in Vietnam: the VISIONARY study. *Ophthalmology* 2014; **121**(11): 2138-46.
19. Glick P, Luoto J, Orrs MS, et al. The individual and household impacts of cataract surgery on older blind adults in ethiopia. *Ophthalmic Epidemiol* 2019; **26**(1): 7-18.
20. Fireison CK, Moore JE. Employment outcomes and educational backgrounds of legally blind adults employed in sheltered industrial settings. *Journal of Visual Impairment and Blindness* 1998; **92**(11): 740-7.
21. Giesen JM, Hierholzer A. Vocational rehabilitation services and employment for SSDI beneficiaries with visual impairments. *Journal of Vocational Rehabilitation* 2016; **44**(2): 175-89.
22. Brown GC, Brown MM, Menezes A, Busbee BG, Lieske HB, Lieske PA. Cataract surgery cost utility revisited in 2012: a new economic paradigm. *Ophthalmology* 2013; **120**(12): 2367-76.
23. Frick KD, Hanson CL, Jacobson GA. Global burden of trachoma and economics of the disease. *The American journal of tropical medicine and hygiene* 2003; **69**(5 Suppl): 1-10.
24. Frick KD, Keuffel EL, Bowman RJ. Epidemiological, demographic, and economic analyses: measurement of the value of trichiasis surgery in The Gambia. *Ophthalmic Epidemiol* 2001; **8**(2-3): 191-201.
25. Haynes R, Gale S, Mugford M, Davies P. Cataract surgery in a community hospital outreach clinic: patients' costs and satisfaction. *Soc Sci Med* 2001; **53**(12): 1631-40.
26. Glewwe P, Park A, Zhao M. A better vision for development: Eyeglasses and academic performance in rural primary schools in China. *Journal of Development Economics* 2016; (122): 170-82.
27. Glewwe P, West KL, Lee J. The Impact of Providing Vision Screening and Free Eyeglasses on Academic Outcomes: Evidence from a Randomized Trial in Title I Elementary Schools in Florida. *J Policy Anal Manage* 2018; **37**(2): 265-300.
28. Hannum E, Zhang Y. Poverty and Proximate Barriers to Learning: Vision Deficiencies, Vision Correction and Educational Outcomes in Rural Northwest China. *World Dev* 2012; **40**(9): 1921-31.

29. Ma X, Zhou Z, Yi H, et al. Effect of providing free glasses on children's educational outcomes in China: cluster randomized controlled trial. *BMJ* 2014; **349**: g5740.
30. Ma Y, Congdon N, Shi Y, et al. Effect of a Local Vision Care Center on Eyeglasses Use and School Performance in Rural China: A Cluster Randomized Clinical Trial. *JAMA Ophthalmol* 2018; **136**(7): 731-7.
31. Hark LA, Thau A, Nutaitis A, et al. Impact of eyeglasses on academic performance in primary school children. *Can J Ophthalmol* 2020; **55**(1): 52-7.
32. Joseph L. Refractive Errors and Academic Achievements of Primary School Children. *Nurs J India* 2014; **105**(6): 269-71.
33. Bruce A, Kelly B, Chambers B, et al. The effect of adherence to spectacle wear on early developing literacy: a longitudinal study based in a large multiethnic city, Bradford, UK. *BMJ Open* 2018; **8**(6): e021277.
34. Baruwa E, Tzu J, Congdon N, He M, Frick KD. Reversal in gender valuations of cataract surgery after the implementation of free screening and low-priced high-quality surgery in a rural population of southern China. *Ophthalmic Epidemiol* 2008; **15**(2): 99-104.
35. Mercer GD, Lyons P, Bassett K. Interventions to improve gender equity in eye care in low-middle income countries: A systematic review. *Ophthalmic Epidemiol* 2019; **26**(3): 189-99.
36. Dickey H, Ikenwilo D, Norwood P, Watson V, Zangelidis A. Utilisation of eye-care services: the effect of Scotland's free eye examination policy. *Health Policy* 2012; **108**(2-3): 286-93.
37. Subzwari S, Desapriya E, Scime G, Babul S, Jivani K, Pike I. Effectiveness of cataract surgery in reducing driving-related difficulties: a systematic review and meta-analysis. *Inj Prev* 2008; **14**(5): 324-8.
38. Owsley C, McGwin G, Jr., Sloane M, Wells J, Stalvey BT, Gauthreaux S. Impact of cataract surgery on motor vehicle crash involvement by older adults. *JAMA* 2002; **288**(7): 841-9.
39. Meuleners LB, Brameld K, Fraser ML, Chow K. The impact of first- and second-eye cataract surgery on motor vehicle crashes and associated costs. *Age Ageing* 2019; **48**(1): 128-33.
40. Meuleners LB, Hendrie D, Lee AH, Ng JQ, Morlet N. The effectiveness of cataract surgery in reducing motor vehicle crashes: a whole population study using linked data. *Ophthalmic Epidemiol* 2012; **19**(1): 23-8.
41. Meuleners LB, Ng JQ, Fraser M, Hendrie D, Morlet N. Impact of gender on first eye cataract surgery and motor vehicle crash risk for older drivers. *Clin Exp Ophthalmol* 2012; **40**(6): 591-6.
42. Schlenker MB, Thiruchelvam D, Redelmeier DA. Association of Cataract Surgery With Traffic Crashes. *JAMA Ophthalmol* 2018; **136**(9): 998-1007.
